# Tactile and Thermal Sensors Built from Carbon–Polymer Nanocomposites—A Critical Review

**DOI:** 10.3390/s21041234

**Published:** 2021-02-09

**Authors:** Chenwang Yuan, Anthony Tony, Ruixue Yin, Kemin Wang, Wenjun Zhang

**Affiliations:** 1 Department of Mechanical Engineering, University of Saskatchewan, Saskatoon, SK S7N 5A9, Canada; chy908@mail.usask.ca (C.Y.); ant359@mail.usask.ca (A.T.); 2 School of Mechanical and Power Engineering, East China University of Science and Technology, Shanghai 200237, China; yinruixue@ecust.edu.cn; 3 School of Mechatronic Engineering and Automation, Shanghai University, Shanghai 200444, China; wangkemin@shu.edu.cn

**Keywords:** carbon–polymer nanocomposite, FCBPSS, tactile sensor, thermal sensor, sensor network

## Abstract

This paper provides a critical review of tactile and thermal sensors which are built from carbon nanomaterial-filled polymer composites (CNPCs). To make the review more comprehensive and systematic, the sensors are viewed as a system, and a general knowledge architecture for a system called function-context-behavior-principle-state-structure (FCBPSS) is employed to classify information as well as knowledge related to CNPC sensors. FCBPSS contains six basic concepts, namely, F: function, C: context, B: behavior, P: principle, and SS: state and structure. As such, the principle that explains why such composites can work as temperature and pressure sensors, various structures of the CNPC sensor, which realize the principle, and the behavior and performance of CNPC sensors are discussed in this review. This review also discusses the fabrication of the CNPC sensor. Based on the critical review and analysis, the future directions of research on the CNPC sensor are discussed; in particular, the need to have a network of CNPC sensors that can be installed on curved bodies such as those of robots is elaborated.

## 1. Introduction

In recent years, industrial robots have emerged both in manufacturing and service environments, having entered an era where humans and robots work together rather than automation alone [1,2]. Emphasis on full automation was realized several decades ago, but humans always tend to be lured by the benefits of automation. This is because these benefits agree well with some traits of humans, such as desires to get the most amount of goods in the shortest time and with the least effort. In fact, these traits are of the short term and are opposite to the notion of sustainability [3,4], a very important concept regarding balanced technological development. In this paper, we define the robot as a kind of machine with a certain level of intelligence as humans and we consider that the robot is constructed by a “wearable” human assistive system (HAS) to augment the robot’s intelligence [2].

One challenge for robots to work intelligently is that they need to know the human’s mind state and behavior, as only then can robots better serve humans and society [2]. This means that the robot is expected to have a good sensing ability. This paper focuses on the sensing of collision between the robot and human, which can be generalized to be a soft tactile and thermal sensor.

An important kind of soft tactile and thermal sensor is where carbon nanomaterials are filled into polymers to form a composite, and this kind of sensor has some excellent properties [5,6]. First, carbon nanomaterials have extraordinary mechanical and electrical properties [7], which provide a solid foundation for sensors. Second, polymers are easy to fabricate, cost-effective, and flexible and of conformance to other entities physically. Third, the material hardness of different polymers, defined as the resistance of a material against a localized surface deformation [8], covers a wide range, which certainly extends the spectrum of applications of such polymer composite sensors [9].

In this paper, we will first propose a framework for classification and analysis of sensors in Section 2, which is based on the general architecture of the ontology of systems called function-context-behavior-principle-state-structure (FCBPSS) [10,11,12]. The salient point of FCBPSS is that it provides a complete system perspective for a subject to be examined—a sensor in this case. We will then employ this framework to classify and analyze various tactile and thermal sensors built from carbon nanomaterial-filled polymer composites in the subsequent sections (Section 3, Section 4, Section 5, and Section 6). Conclusions and future directions will be presented in Section 7.

## 2. FCBPSS Framework for Classification and Analysis of Sensors

**Figure 1:**
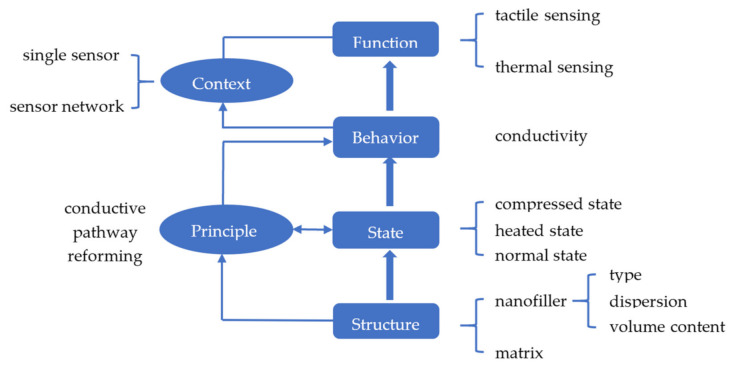
The framework of carbon nanomaterial-filled polymer composites.

The function-context-behavior-principle-state-structure (FCBPSS) is a general architecture of ontology of any system, developed by Zhang and Lin [10,12,13] and Zhang and Wang [11]. It has six categories of concepts [11]: structure, state, behavior, principle, context, and function (see Figure 1). According to Zhang and Lin [10], “Structure is a set of entities connected in a meaningful way”. States are indicators of the presence and properties of an entity in a system, adapted from Zhang and Wang [11]. There could be many states of an entity at a point in time, space, and an event, and therefore a representation approach of class instance or variable value is applied to the state concept, resulting in the concept of the state variable. With the concept of the state variable, a particular state (e.g., the mass of an entity is 50 g) is represented by a notation such as “Mass = 50 g”, where “Mass” is a state variable, and “50 g” is a value of the state variable. According to Zhang and Wang [11], “Behavior is the causal relationship among a set of state variables.” The principle of the system governs the behavior; the principle is deep knowledge of the causal relationship.

By considering carbon nanomaterial-filled polymer composites (CNPCs) as a system, this system can be represented with FCBPSS. In Figure 1, the structure of the system refers to the nanofiller and the matrix along with the type, content, and distribution of the nanofillers. It is clear that the structure will affect the sensing performance measured by the so-called gauge factor. The working principle or principle that governs how the CNPC works is conductive pathway reforming (Figure 1). For both tactile and thermal sensors, the CNPC has three levels of the overall condition state: normal, heated, and compressed. Regarding the behavior of the system (the CNPC system in this case), first, different levels of the overall condition state imply different conductive pathways and thus different conductivity changes in the CNPC. Second, the behavior in this case refers to the relationship between a tactile or thermal stimulus to the CNPC and the overall condition state and thus the conductivity. The context of the system is such that a single CNPC can work as a tactile or thermal sensor if it is further connected in a circuit, and a set of CNPC sensors can form a sensor network for a wide range of a target system to be sensed and monitored. Finally, the function of the system refers to the performance of the system, which determines the usefulness of the system. In the following, Section 3 will discuss the principle of the CNPC, Section 4 will discuss the structure and states of the CNPC, and Section 5 will discuss the performance of the CNPC.

## 3. The Principle of the CNPC Sensor

There are two working principles or principles of the CNPC sensor. The first principle is conductive pathway reforming [14]. The nanofillers are dispersed in the polymer matrix and form filler networks that have many pathways under the condition that the volume content of nanofillers is higher than a particular value (or threshold) according to percolation theory [15,16,17,18]. The conductivity of the whole system is based on the pathways. When the external pressure stimuli or temperature stimuli are applied to the system, the pathways change and thus the conductivity changes. It was noted by Miao et al. [14] that pathways, in particular CNPCs, depend on particular fabrication techniques. The second principle is electron tunneling or hopping. This principle states that when the distance between neighboring carbon nanofillers reaches a critical value, the electrons on the outer layer of carbon atoms are able to hop and move to form conductive pathways. To a particular CNPC, both principles may play their roles, but in the case of CNPCs, the first principle dominates. It was also noted that by increasing the volume content of carbon nanofillers of a CNPC after reaching the percolation threshold, its conductivity increases [19], which seems to be obvious, but there seems to be a limit of the increase according to the work of Miao et al. [9].

Both tactile and thermal sensors can be developed based on the principle of conductive pathways reforming. For the tactile sensing, the pressure is applied on a CNPC, which causes the change in pathways in a CNPC and the subsequent change in the conductivity or resistance. For thermal sensing, the heat is applied to the CNPC, which causes two changes in the structure of the CNPC. The first change is that the electrons at the outer layer of carbon atoms become more active due to the increase in the temperature of individual nanofillers. This further causes the decrease in the resistance of the CNPC. The second change is the expansion of the whole CNPC system due to the thermal expansion law, and this may subsequently reduce the number of pathways and eventually may increase the resistance of the CNPC.

**Figure 2:**
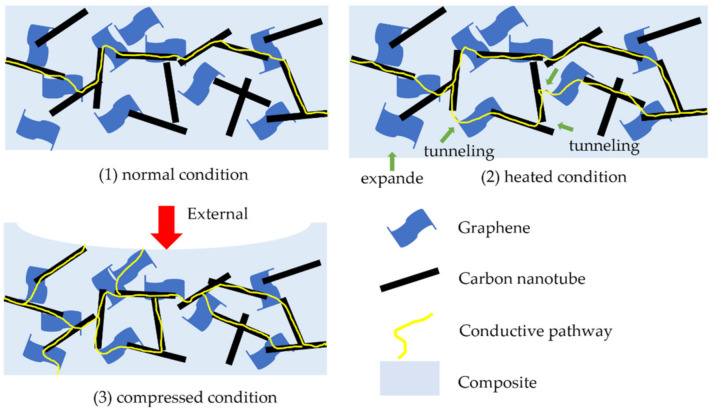
Three conditions of nanocomposites: (**1**) normal condition; (**2**) heated condition; (**3**) compressed condition.

As mentioned before, there are three levels of the overall condition state for the CNPC associated with tactile and thermal sensing: normal, heated, and compressed (as shown in Figure 2). For the clarity of the subsequent discussions, in this paper, the levels of the overall condition state are called conditions. At the normal condition, the CNPC has its state and properties without any contribution from the external force nor heating source. At the heated condition, the CNPC has its elevated state and properties with a contribution from the heating flux over the CNPC. At the compressed condition, the CNPC has its elevated state and properties with a contribution from pressures over the CNPC. At the normal condition, the composite is conductive when the volume content of carbon nanofillers is higher than the percolation threshold. From the normal condition to the compressed condition, the conductivity of nanocomposites would increase as the result of forming more conductive pathways. This is because the force applied to the CNPC can cause deformations of the CNPC and can thus change the formation of conductive pathways.

From the normal condition to the heated condition, the resistance of the CNPC would change. However, the resistance change due to the change in temperature depends on several factors, namely, the type, content, dispersion of nanofillers, and properties of polymer matrixes. It is to be noted that the mechanism governing the temperature resistance effect is still unclear. Currently, there are three mechanisms or principles, namely, (1) the tunneling effect mechanism [20], (2) the thermal expansion mechanism [21], and (3) the nanofiller rearrangement mechanism [22]. The tunneling mechanism states that the increased temperature could induce more tunneling and thus create more conductive pathways, which means that the temperature–resistance relationship shows a negative temperature coefficient (NTC) behavior. Xi et al. [20] presented this relationship as follows:

(1) $$R\left(T\right)=R_{0}\exp\left[\frac{T_{1}}{\begin{array}{ccc} T_{0} & + & T \end{array}}\right]$$

where $T_{0}$ is the temperature, below which the elastic tunneling conduction dominates, $T_{1}$ is the temperature, at which the electron could gain enough energy to hop, R is the resistance, and $T_{0}$, $T_{1}$, and $R_{0}$ are fitting parameters. Neitzert et al. produced a multi-walled carbon nanotube (MWCNT)/epoxy composite and used it as a temperature sensor as well as an electrical heating element [23]. They conducted experiments and showed that the resistance–temperature relationship of the MWCNT/epoxy composite fits well with Equation (1).

**Figure 3:**
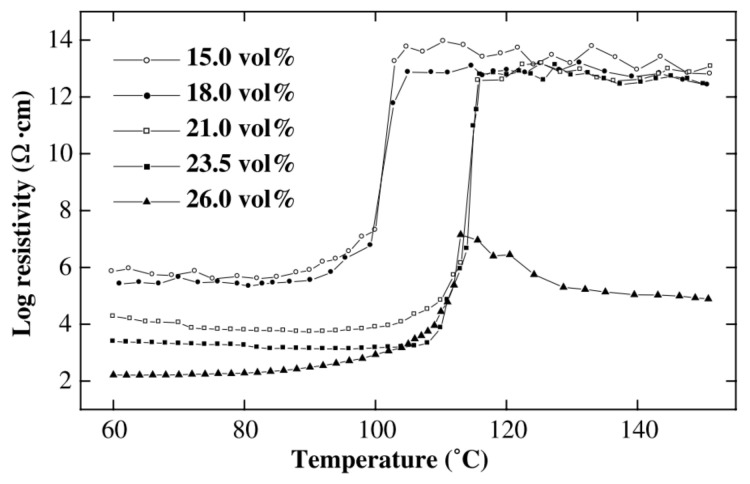
Log resistivity against temperature of low-molecular weight polyethylene (LMWPE)/ultra-high molecular weight polyethylene (UHMWPE) composites filled with different carbon fiber volume contents [21]. Reproduced with permission.

However, more studies showed a positive temperature coefficient (PTC) phenomenon of the CNPC. Regarding the thermal expansion mechanism, when the CNPC is heated, the thermal expansion coefficient of polymers is much higher than that of carbon nanofillers, which could increase the distance of neighboring nanofillers and reduce conductive pathways, resulting in higher resistance. Xi et al. prepared ultra-high molecular weight polyethylene (UHMWPE) and low-molecular weight polyethylene (LMWPE) composites filled with short carbon fibers and conducted experiments. The result of their experiments showed that the maximum PTC effect of the carbon fiber-filled LMWPE/UHMWPE composites can lead to an increase of up to nine orders of magnitude when the volume content of the carbon fiber is 23.5% (see Figure 3) [21]. Bao, Liang, and Tjong compared an MWCNT-filled polypropylene nanocomposite (MWCNT/PP) and an MWCNT- and montmorillonite-filled polypropylene nanocomposite (MWCNT/MMT/PP) [24]. They also found the PTC phenomenon on both the MWCNT/PP and MWCNT/MMT/PP composites. However, as the addition of montmorillonite increases the viscosity of the MWCNT/MMT/PP composite, it restricts the movement of polypropylene molecules and thus decreases the loss of conductive pathways (hence the PTC effect).

Regarding the mechanism of nanofiller rearrangement, it states that changes occur in the gathering and/or orientation of nanofillers when the polymer matrix is melted by the increased temperature [22]. Ferrara et al. investigated several properties of a CNT/linear low-density polyethylene (LLDPE) composite when applying an electric field [22]. They found the resistance of the composite increases when heated and they explained this PTC phenomenon as the result of the regrouping, gathering, and/or orientation of CNTs in the melt polymer. According to their experiments, the temperature–resistance relationship may also be affected by the composite’s crystallinity and the voltage applied on the composite.

**Figure 4:**
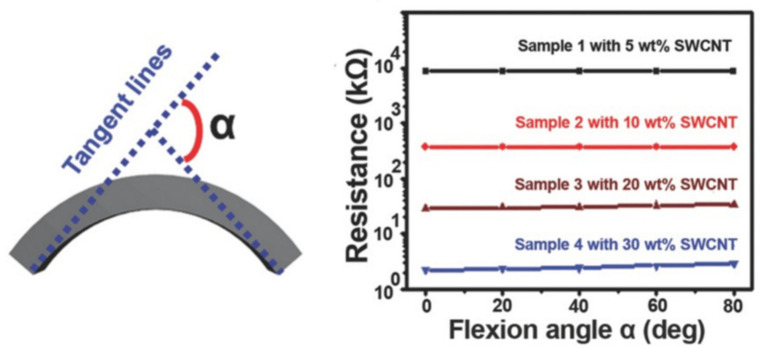
The definition of flexion angle α and the resistance change upon increasing α [25]. Reproduced with permission.

Based on the principles discussed above, different tactile and thermal sensors have been developed. However, considering that both deformation and heat could lead to a resistance change, the performance evaluation of a tactile or thermal sensor is usually conducted in an univariant environment that only has force or heat applied. This result could help calibrate the sensing ability of the tactile sensor in a specific temperature or the thermal sensor in a specific shape. It is hard to distinguish the resistance change caused by deformation or heat, but measures could be taken to eliminate the influence. Yang et al. developed a soft thermal sensor based on a single-walled carbon nanotube (SWCNT)-filled polymer composite and measured the resistance change caused by deformation of the thermal sensor [25]. By increasing the flexion angle α from 0° to 80° (see Figure 4), the resistance of the thermal sensor with 20 wt% of SWCNT increased from 20 to 24 kΩ. When there is no deformation, the resistance of the thermal sensor decreases from 29 to 11 kΩ, with the temperature increasing from 0 to 80 °C. Comparing the resistance change caused by the deformation and temperature change, the deformation has a relatively significant influence on the function of the thermal sensor. Therefore, it is better to give specific calibration values for tactile sensors in specific temperature or thermal sensors in specific shapes to avoid the influence.

In summary, tactile and thermal sensing can be quantified by the resistance change in the CNPC, and the resistance change is governed by conductive pathway reforming. For tactile sensing, the pressure applied to the CNPC causes the deformation of the CNPC, and the deformation causes the reforming of conductive pathways—particularly the increase in the resistance. For thermal sensing, the temperature change causes the whole volume change due to the thermal expansion law, particularly the increase in the resistance, and the activeness of electrons, which further causes the reforming of conductive pathways but is uncertain concerning the increase or decrease in the resistance.

## 4. Structure and State of the CNPC

A CNPC contains a matrix and nanofillers. Carbon nanofillers have different structures, mainly classified into carbon black, fullerenes, carbon nanotubes, and graphene. Due to the outstanding electrical and electronic properties and large aspect ratio, carbon nanotubes and graphene are widely used in polymer nanocomposites to tailor to different applications. Carbon nanotubes can be viewed by rolling graphene sheets. Single-walled carbon nanotubes (SWCNTs) are tubes that are rolled by one graphene sheet, and multi-walled carbon nanotubes (MWCNTs) are tubes that are rolled by multiple graphene sheets.

The properties of a CNPC are based on the nanostructure of the composite, which is composed of a matrix and nanofillers. For the carbon nanofiller, its type, dispersion condition, and volume content will affect the electrical and thermal properties. Further, different polymers used as the matrix would also have different mechanical, electrical, and thermal properties.

### 4.1. The Types of Carbon Nanofillers

**Figure 5:**
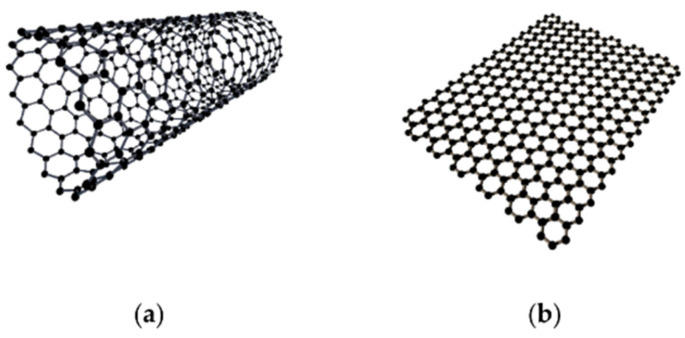
Structure of carbon nanotube (**a**) and graphene (**b**) [30]. Reproduced with permission.

Carbon nanotubes (1D) and graphene (2D) are most widely used as carbon nanofillers, and they have different effects on the properties of CNPCs (see Figure 5). Table 1 and Table 2 show the differences between carbon nanotubes and graphene. The aspect ratio of graphene is around 58% higher than the aspect ratio of MWCNTs because of the 2D structure of graphene. The large aspect ratio of graphene could maximize its contact area with the polymer matrix, which helps transfer stress between the graphene and polymer matrix [26]. The large contact area could also improve the properties compared with polymer composites filled with CNTs. However, the large surface area would also cause aggregation and bundling of graphene sheets because of the large van der Waals force. Aggregation of nanomaterials would further influence the properties of the composites, such as the electrical conductivity and thermal conductivity. Aggregation needs to be avoided in fabrication, to which use of surfactants [27], functionalization of carbon nanotubes [28], and physical stirring [29] are the most commonly used methods.

CNTs have a relatively low aspect ratio and low surface area, compared with graphene. Some researchers reported a better performance of polymer nanocomposites filled with mixed CNTs and graphene [26,33]. Zhang et al. proposed the so-called hybridization design principle, which has two principles: the complementary principle and the compatibility principle [34]. For the hybridization of CNTs and graphene, a model for quantitative analysis is available in the literature. As CNTs and graphene have similar electrical and thermal properties, it is difficult to tell which one contributes more to composites with a mixture of nanofillers. Some researchers considered the conductivity improvement of mixed CNT and graphene nanocomposites as the result of forming a 3D mixture [35]. As the CNT is a 1D material and graphene is a 2D material, the CNT and graphene would form a 3D mixture. The mixture of CNT and graphene could enjoy the advantages of each other with some specific treatments.

Table 3 lists several existing studies on mixtures of carbon nanotubes and graphene as the nanofiller in the polymer matrix.

Per Table 3, the electrical conductivity and percolation threshold can be compared when adding different carbon nanofillers into the same polymer matrix. Yang et al. argued that graphene platelets could maximize the stress transfer between the polymer matrix and nanofillers due to its large contact area [26]. However, as opposed to the sole filling of carbon nanomaterials, a CNPC filled with a mixture of CNTs and graphene presents better conductivity.

Yang et al. explored the synergetic effects of filling graphene platelets, which are platelet-like graphite nanocrystals with multiple graphene layers, and carbon nanotubes on the mechanical and thermal properties based on epoxy composites [26]. They argued that graphene platelets could help maximize the stress and heat transfer between the polymer matrix and nanofillers due to its large contact area. Thus, graphene platelets could provide better reinforcement for a CNPC compared with CNTs. However, the large contact area would cause a large van der Waals force and result in the aggregation and stacking of graphene sheets. Therefore, they examined the effects of mixing 2D graphene platelets with 1D multi-walled carbon nanotubes, from which they expected to find a new way to overcome the aggregation problem as the MWCNTs could separate the graphene sheets and increase the contact area at the same time.

**Figure 6:**
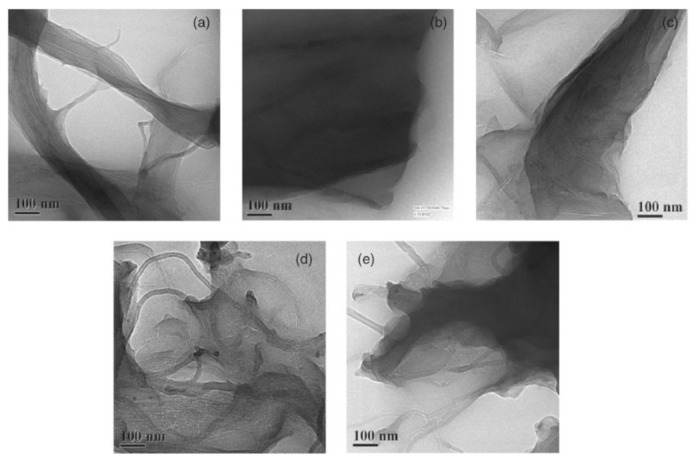
TEM images of (**a**) MWCNT (0.375 wt%)/silicone rubber (VMQ), (**b**) graphene (0.375 wt%)/VMQ, (**c**) MWCNT–graphene (0.375 wt%)/VMQ, (**d**) MWCNT–graphene (0.75 wt%)/VMQ, and (**e**) MWCNT–graphene (1.5 wt%)/VMQ [49]. Reproduced with permission.

Pradhan and Srivastava investigated the synergistic effect of an MWCNT–graphene nanofiller in enhancing the properties of silicone rubber (VMQ) [49]. They fabricated an MWCNT/VMQ nanocomposite, a graphene/VMQ composite, and an MWCNT–graphene/VMQ nanocomposite. The mixture filler was made with the MWCNT and graphene at a 1:1 weight ratio. Figure 6 shows the transmission electron microscopy (TEM) images of the MWCNT (0.375 wt%)/VMQ composite, the graphene (0.375 wt%)/VMQ composite, the MWCNT–graphene (0.375 wt%)/VMQ composite, the MWCNT–graphene (0.75 wt%)/VMQ composite, and the MWCNT–graphene (1.5 wt%)/VMQ composite [49]. According to Figure 6a,b, MWCNTs are entangled into bundles and graphene nanosheets are shown to be stacked in the composite. In Figure 6c–e, the MWCNT–graphene (1:1) mixture shows a better dispersion. Especially for Figure 6d, when the MWCNT–graphene is at 0.75 wt%, the graphene nanosheets are separated by MWCNTs and MWCNTs are attached on the surface of the graphene. Figure 6d shows a 3D structure formed by a 1D MWCNT and 2D graphene. Pradhan and Srivastava further studied the mechanical and thermal properties of the MWCNT/VMQ nanocomposite, graphene/VMQ composite, and MWCNT–graphene/VMQ nanocomposite. They concluded that the synergistic effect of the MWCNT–graphene 3D structure could account for the improvement in mechanical and thermal properties [49].

Punetha et al. also concluded that the mixing of one-dimensional CNTs and two-dimensional graphene could form a three-dimensional mixture that can solve the problem of dispersion of the sole nanofiller and provide synergistic properties compared with composites with a sole nanofiller [33]. In summary, the type of carbon nanomaterials added into the polymer composite has direct effects on the properties of the composite. Mixed filling is promising to achieve better performances with different nanostructures.

### 4.2. The Distribution of Carbon Nanofillers

The distribution of carbon nanofillers has a direct impact on the formation of conductive pathways. Aggregation of nanofillers could lower the composite’s conductivity. Aggregation of nanofillers is caused by the intrinsic van der Waals forces. To overcome the trend of aggregation and to make the nanofillers disperse homogenously, there are several solutions reported in the literature, including ultrasonication, high-shear mixing, surfactants, alignment, chemical modification, and polymer chain wrapping [50]. Zhu et al. found that a homogenous distribution is important not only for electrical properties improvement but also for mechanical properties enhancement [50]. Grossiord et al. pointed out that good distribution in terms of uniformity could lead to more conductive pathways, which could further lead to good conductivity [27].

However, some researchers hold different opinions that aggregation has positive effects on increasing conductivity. Delozier et al. thought that a good uniform distribution would lower the possibility that carbon nanofiller bundles would connect to each other to form conductive pathways [51]. However, this explanation is not convincing as the aggregation of nanofillers may not be able to connect neighboring bundles. Du et al. proposed that electrical conductivity would be increased by heterogeneous distributions of SWNTs instead of uniform distributions [52].

Besides qualitative analysis, Li et al. provided an equation to describe the relationship between the distribution of carbon nanofillers and the conductivity of the composite [53]. They proposed that the percolation threshold of the composite can be represented by

(2) $$P_{c}=\frac{\delta \varepsilon \pi }{6}+\frac{\left(1-\delta \right)27\pi d^{2}}{4l^{2}}$$

where $P_{c}$ means the percolation threshold of the composite, δ means the volume fraction of the aggregated CNTs, ε means the volume content of CNTs in an aggregation, d means the diameter of the CNTs, and l means the length of the CNTs. However, this equation remains to be proved by more experiments. In summary, uniformity in the distribution of nanofillers over polymer matrixes is important and has some great effect on the conductivity of the composites. More quantitative analysis needs to be explored in the future.

### 4.3. The Volumn Content of Carbon Nanofillers

In addition to the distribution of carbon nanofillers, the quantity of nanofillers is also a critical parameter that affects the properties of the CNPC. An increase in volume content would make the composite become conductive after achieving the percolation threshold. From Table 3, it can be seen that for the same matrix and fabrication method, different weight mixing ratios of CNTs and graphene would affect the electrical conductivity of the composite, which would consequently affect the sensing performance of the tactile sensor.

Stauffer et al. presented a predictive relationship based on the percolation threshold [54]:

(3) $$\rho =\rho_{0}{\left(\nu -\nu_{c}\right)}^{t},$$

where ρ is the composite resistivity, $\rho_{0}$ is the resistivity of the conductive filler, ν is the volume content of the filler, $\nu_{c}$ is the percolation threshold of the filler, and t is the critical exponent.

**Figure 7:**
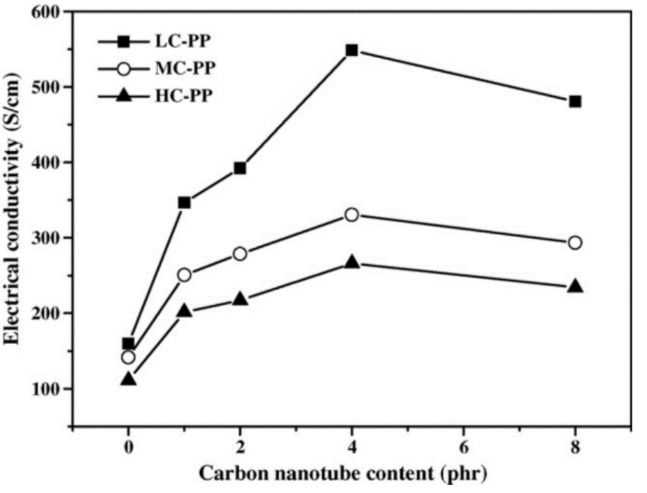
The bulk electrical conductivities of the nanocomposite bipolar plates with various MWCNT contents [59]. Reproduced with permission.

Based on experiments, Regev et al., Martin et al., Sandler et al., and Hu et al. reported that the critical exponent value ranges from 0.7 to 3.1 [55,56,57,58]. However, this predictive relationship is only suitable for a small amount of carbon nanofillers added into the polymer composite. When the volume content of carbon nanofillers is relatively high (compared with the percolation threshold), large bundles and aggregations would appear and the conductivity of composites tends to be leveled off, or even decreased [59]. Liao et al. conducted experiments on nanocomposite bipolar plates by filling different volumes of MWCNTs into high-crystallinity polypropylene (HC-PP), medium-crystallinity polypropylene (MC-PP), and low-crystallinity polypropylene (LC-PP) and tested their conductivities, as shown in Figure 7 [59]. All three composites show the same trend of a conductivity decrease when filled with higher amounts of carbon nanotubes.

Engel et al. reported the relationship between the MWCNT loading and the resistance of an MWCNT-filled conductive elastomer [60]. They found that when the nanofiller’s loading content is near the percolation threshold, the resistance of the MWCNT-filled conductive elastomer has a sensitive response to deformation, which means that the conductive elastomer gets the best tactile sensitivity but with a nonlinear response and noise as well. When the nanofiller’s loading content is much higher than the percolation threshold, its response sensitivity to deformation goes down but presents better linearity. In summary, the volume content of carbon nanofillers could affect the conductivity of the composite directly. An appropriate volume content needs to be chosen based on experiments.

### 4.4. The Type of Polymer Matrixes

Various kinds of polymers have been tried as the matrix of CNPCs. Based on the molecular forces, polymers are classified into four types, elastomers, fibers, thermoplastics, and thermosets. The molecular force in elastomers is the weakest among the four types, which enables elastomers with good stretchability. Thermoplastics and thermosets are two main categories of polymers that are commonly used in plastic products. Thermoplastics become soft when heated and can be molded to obtain a desired shape. The molecular force in thermoplastics is the van der Waal force. However, thermosets become rigid and infusible when being heated. They have low molecular masses compared with thermoplastics, which are long-chain polymers. The difference between thermoplastics and thermosets is that the repeated heating and cooling for the shaping process of thermoplastics is possible while impossible for thermosets. The intrinsic reason is that thermoplastics would not contain any cross-bond, but thermosets would form cross-links between polymer chairs and form a 3D structure when heated. As polymers work as the substrate of tactile/thermal sensors, the properties that could affect the performance of a tactile/thermal sensor are Young’s modulus and the thermal expansion coefficient. Based on the same polymer substrate, various tactile/thermal sensors could be fabricated when filling different types and content ratios of carbon nanomaterials. However, there are two general notes that may help researchers choose a suitable polymer as the substrate: (1) polymers with a smaller Young’s modulus would help form a tactile sensor with higher sensitivity; (2) polymers with a bigger thermal expansion coefficient would help form a thermal sensor with higher sensitivity if the thermal expansion mechanism is the governing mechanism. Table 4 provides general notes for the suitable application of different polymers as the sensor matrix. Further, Table 5 provides the electrical percolation thresholds for CNT-filled thermoplastic and thermoset polymers.

From the perspective of safety for humans and the environments in, for example, human–robot–environment systems, two types of polymers are receiving increased attention, that is, biocompatible and biodegradable polymers. Biocompatibility is a measure of negative effects of materials on humans and environments, see [61,62,63,64,65,66]. Biodegradability is a measure of decomposition of a polymer into elements and compounds [67,68]. Poly (lactic acid) (PLA), poly (glycolic acid) (PGA), and poly(3-caprolactone) (PCL) are the most widely used biodegradable synthetic polymers [69]. PLA is a hydrophobic material and could maintain mechanical properties for several months before degradation. Guo produced a biodegradable polymer nanocomposite based on a PLA matrix and tested various mechanical properties [70]. In contrast, PGA is hydrophilic and degrades faster than PLA. PGA would lose mechanical properties between two and four weeks. Adding carbon nanomaterials as the filler would improve the mechanical and electrical properties of biocompatible and biodegradable polymers and could promote the development of tactile and thermal sensing applications.

For both tactile and thermal sensors, the polymer matrix performs as an agent to transform the deformation and temperature change to the resistance of the CNPC. Therefore, the properties of polymers have dominant effects on the sensitivity of the composites. A soft and thermal-sensitive transformation polymer should contribute to better sensitivity as the deformation or temperature change caused by external factors is more quickly transferred to resistance change [37,39].

## 5. The Performance of the CNPC Sensor and Sensing System

CNPC sensors work in two different contexts: a single sensor and a sensor network. The performances of a single CNPC sensor and a sensor network are measured by key indexes such as the gauge factor, linearity, repeatability, response and recovery time, and durability. Considering that the gauge factor is the most important performance index and is readily available, this paper only concerns the gauge factor.

Many efforts have been spent on developing single tactile or thermal sensors. Besides functioning as a single sensor, a sensor network is another effective way to obtain sensing ability. Table 6 lists several tactile sensing systems with their structures and performances and Table 7 provides several thermal sensing systems. Selected systems are commented on below.

**Figure 8:**
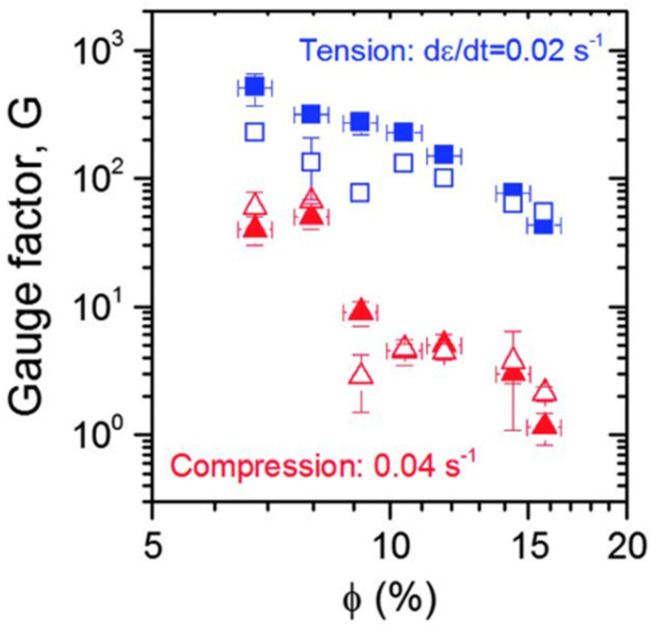
Gauge factor (G) plotted versus volume fraction (∅) for both tensile (blue) and compressive (red) measurements. The solid symbols represent measured values, and the open symbols represent predicted values [71]. Reproduced with permission.

Among the tactile sensing systems in Table 6, there are two tactile sensors that have a relatively high gauge factor. One was fabricated by Boland et al. They embedded graphene in a highly viscoelastic silicone polymer and obtained a tactile sensor with a gauge factor of > 500 that can even detect a spider’s footsteps on it [71]. The gauge factor reaches 535 for tensile measurement when the volume content of graphene is 6.8% (see Figure 8). The polymer matrix they used was a lightly cross-linked silicone polymer, which is commonly known as Silly Putty. The graphene nanofillers form a mobile conductive network that could easily breakdown and reform when the matrix is deformed. However, this highly sensitive tactile sensor only works at a limited strain range of 0–2%. This strain range limits its application for light force detection. Another tactile sensor that has a high gauge factor was fabricated by He et al. Their tactile sensor achieves a gauge factor of approximately 2800 in the strain range of 5–100% [80]. However, the problem with He’s sensor is that there is hysteresis of the resistance change with strain higher than 20%. The hysteresis would influence the repeatability of tactile sensing. For tactile sensors that have a relatively high gauge factor, a limited strain range and poor repeatability are two typical disadvantages.

**Figure 9:**
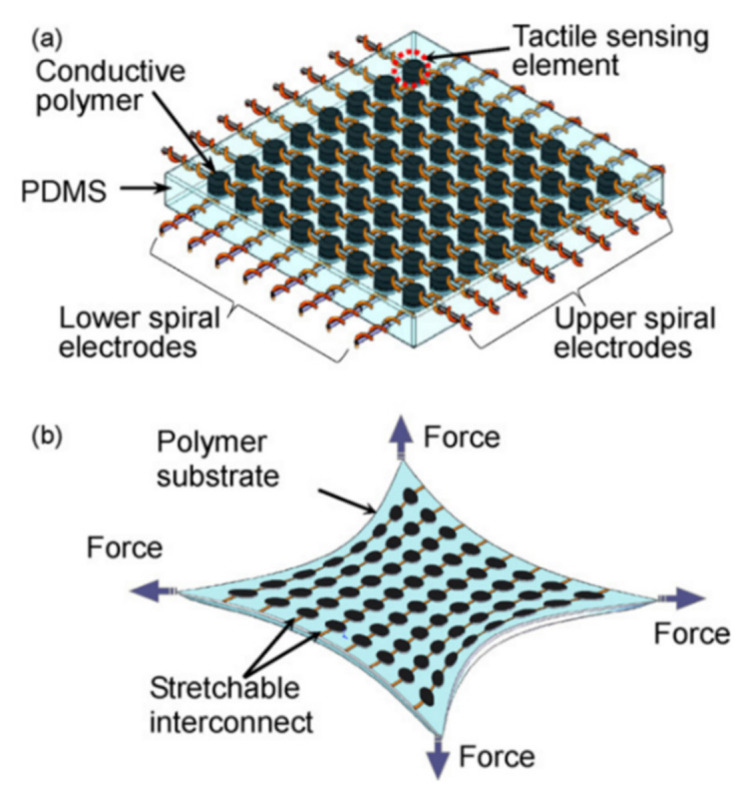
(**a**) The schematic of a tactile sensing array; (**b**) the sensing system under stretching [82]. Reproduced with permission.

For the structure of tactile sensing systems, a single sensor has limited coverage. Researchers explored different sensor networks based on tactile sensors. Cheng et al. developed a tactile sensing array by using conductive polymers filled with carbon nanomaterials, see Figure 9. Through forming a sensor network, the sensing system obtains more sensing ability, e.g., twist force and irregular distribution pressure [82]. However, this tactile sensing system has poor linearity of resistance versus pressure and resistance versus twisting angle. This poor linearity may be attributed to the sensor network structure as different parts of the structure have a nonlinear deformation response to pressure and twisting.

Sun et al. produced 6 × 8 tactile sensor networks based on a multi-walled carbon nanotube (MWCNT)-filled polydimethylsiloxane (PDMS) composite [78]. The composite shows high sensitivity in the low-pressure range (<300 Pa) and the sensor network can work stably in the temperature range of −20 to 50 °C. Similar to Cheng’s tactile sensing system, Sun’s 6 × 8 tactile sensor arrays also have poor linearity of resistance versus pressure. In addition, in the low-pressure range (100–300 Pa), there is a significant hysteresis of resistance change despite the high sensitivity performance.

Chang et al. fabricated a piezo resistance stretchable pressure sensor based on reduced graphene oxide and a VHB elastomer for surgical robots [75]. The novel part of their work is based on the wrinkle architecture of the sensor, which is inspired by the skin of the Shar Pei dog. This wrinkle architecture could be understood as pre-compressed, which reserves the space for stretching. Another advantage is that the stretching and loading operation could change the resistance of the tactile sensor in opposite directions. This could help distinguish the tensile and compressive stress. They applied this tactile sensor on surgical robots and proved its sensing ability. However, this tactile sensor was in the shape of a film and could not locate the position of where the strain was created.

Chen et al. proposed a touch-sensing skin for collaborative robots [83]. This skin was fabricated by coating graphite-filled latex on rubber and has a piezoresistive feature. They applied this tactile sensing skin on collaborative robots with five different paddings. As a conclusion, foam is considered as the best padding material working with this tactile sensing skin on collaborative robots. It was a great idea to view tactile sensing skin and padding as a system and test their performance. However, only bonding tactile sensing skin with padding physically is far from enough, and well-designed physically and functionally bonding is still expected.

Based on the analysis above, there are several factors that may influence the performance of the tactile sensing system. The content ratio of carbon nanomaterials, the structure of a tactile sensing system, and Young’s modulus of the polymer matrix all have impacts on the performance of the sensing system. The design of a specific tactile sensing system is a procedure of tuning between these factors. For example, a softer polymer matrix may lead to a more sensitive tactile sensor but would also limit its strain range.

Table 7 provides several thermal sensing systems. Different from tactile sensing systems, thermal sensing does not have a significant desire for a larger coverage. Most of the thermal sensors work alone.

**Figure 10:**
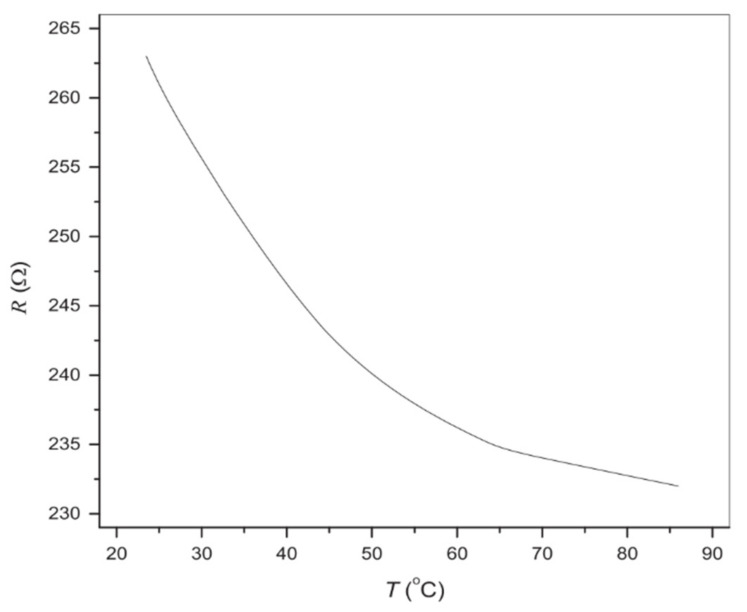
Resistance and temperature relationship of the CNT–GMSA composite [85]. Reproduced with permission.

Karimov et al. fabricated a temperature gradient sensor based on a CNT composite [85]. They proved that the conductivity change in the CNT composite temperature gradient sensor is attributed to the percolation theory. Figure 10 shows the resistance change when the temperature increases from 25 to 85 °C. It shows good linearity of the resistance–temperature gradient between 25 and 45 °C and could be used for human body temperature sensors considering its linearity range. The sensor shows an NTC which may be related to its relatively high content ratio of CNTs. One explanation is that the CNTs are crowdedly connected to each other in the composite. Once heated, the thermal expansion cannot disconnect many conductive pathways, while the tunneling mechanism increases conductive pathways and decreases the contact resistance of the connected CNTs. Therefore, the tunneling mechanism is governing the temperature resistance effect and overall shows a negative temperature coefficient. Another thermal sensor that shows an NTC was fabricated by Yang et al. [25]. It also has a relatively high content of MWCNTs (20 wt%). This high content ratio coincides with our explanation for the cause of an NTC. This explanation coincides with the numerical simulation conducted by Alamusi et al. [90]. Regarding the governing mechanism of the temperature resistance effect, future work is expected to explore the intrinsic principle of the mechanisms.

However, Lamberti et al. and Neitzert et al. fabricated thermal sensors that show an NTC while the content ratio of MWCNTs is only at 1 [87] and 0.5 wt% [23]. They both used DiGlycidil-Ether Bisphenol-A/4,4-diaminodiphenyl sulfone epoxy as the matrix. As discussed in Section 3, there are three mechanisms that may be working at the same time for the temperature resistance effect and the governing mechanism would decide whether the temperature resistance effect is a PTC or an NTC. A higher content ratio and better homogeneous distribution of carbon nanomaterials would favor the tunneling mechanism as thermal expansion would disconnect a significant number of conductive pathways. Another factor is the thermal expansion coefficient of the polymer matrix. A polymer matrix with a higher thermal expansion coefficient would favor the thermal expansion mechanism for the temperature resistance effect.

For the thermal sensor made by Lamberti et al., the presence of an NTC with 1 wt% MWCNTs may be related to the hydrotalcite clay. Here, 0.7 wt% of hydrotalcite clay was added to the MWCNTs. The lamellar shape of the clay favors the deposition of MWCNTs over its surface and therefore forms a better dispersion of MWCNTs [87]. Although the content ratio of MWCNTs is not as high as the thermal sensor from [85] and [25], the homogeneous distribution of MWCNTs may lead to the failure of the thermal expansion mechanism. This is because MWCNTs are distributed evenly in the composite that even the 1 wt% MWCNT load could fully connect MWCNTs and keep them connected when thermal expansion happens. Therefore, the tunneling mechanism is governing the temperature resistance effect and shows an NTC. The better dispersion of MWCNTs also leads to properties improvement, including the thermal sensing ability. It has a linear temperature resistance range between 30 and 110 °C. The thermal sensor made by Neitzert et al. has a larger temperature range from room temperature to 150 °C [23], but the linearity of the temperature resistance is not good.

Alamusi et al. reported a thermal sensor that shows a PTC [86]. It has a rate of resistivity change with a temperature of 64 Ω m K^−1^ in the range of 330 to 375 K. It shows good linearity, but the working range is limited for application. Based on the analysis above, for a thermal sensor, the content ratio and distribution of carbon nanomaterials and the thermal expansion coefficient of the polymer matrix all have an impact on the sensing ability. Different parameters may lead to a different temperature resistance effect, but it would be better to have one mechanism as the governing mechanism to obtain a linear temperature resistance effect.

For these different kinds of sensor networks, there are several essential parameters that are used to compare their performances, including sensitivity, linearity, response and recovery time, and durability [91]. Based on different structures, filler types, and polymers, sensor networks would have advantages in some parameters and disadvantages in other parameters. In summary, tactile and thermal sensors built from carbon nanomaterial-filled polymer composites are widely used in robotics, human–machine interaction [92], biomedical application [93], and wearable electronics [94]. Single sensors or sensor networks are utilized in different contexts. The forming of sensor networks is a more effective way to expend the sensing area and capacity.

## 6. Fabrication Techniques

The fabrication procedure has important effects on the formation of conductive networks, which will contribute to the conductive performance of CNPCs [95]. The fabrication technique of CNPCs mainly has three different methods. Table 8 provides advantages, disadvantages, and notes of different fabrication methods.

(1) Solution method [38]: Prepare a polymer solvent and dissolve nanofillers into the polymer solvent. After sufficient dissolution, evaporating the water of the solvent then results in a polymer nanocomposite, which is in the matrix form. This is the most commonly used method.
(2) Melt mixing method [46]: Prepare a polymer solvent and add the nanofillers directly into the solvent. After solidification of the polymer solvent nanocomposite, the polymer nanocomposite is obtained. This method seems easier than the solution method; however, the biggest problem is that it is difficult to disperse the nanofillers in a random and uniform way. This means that the nanofillers may aggregate in a small area, which produces some unexpected mechanical and physical properties in the resulting composite.
(3) In situ polymerization method [41]: This method is different from the foregoing methods in such a way that the polymer composite is formed with polymerization at the same time. In particular, it uses a monomer solution or a liquid monomer to dissolve nanofillers and then to polymerize the monomer to form the polymer composite.

**Figure 11:**
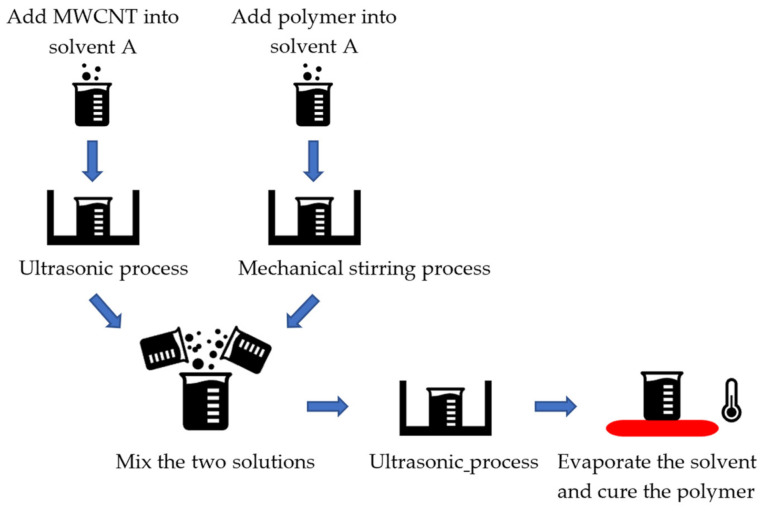
Solution method with the ultrasonic process.

Aggregation of nanofillers in the fabrication process occurs in all the foregoing methods, as this is due to the inherent property of nanofillers, i.e., high surface free energy. Efforts have been taken to develop methods to overcome this problem. The ultrasonic process [96] and addition of surfactants [9] are the two most commonly used methods in this regard. Figure 11 shows a typical procedure of the ultrasonic process in the solution method. MWCNTs and a polymer resin are dispersed in the same solvent and mixed. The ultrasonic process is then employed to help disperse the MWCNTs in the solution. After the ultrasonic process, the polymer is cured, resulting in an MWCNT-filled polymer composite. In this ultrasonic process, the choice of the solvent is extremely important [97]. Different organic solvents have been examined, including dimethylformamide [98], chloroform [99], tetrahydrofuran [100], and toluene [101]. To find out which one works better with MWCNTs, Liu et al. investigated the four organic solvents and found that chloroform beats the other three [97].

The above special treatments may be called the mechanical method. The chemical method may make sense, which is based on functionalization of carbon nanomaterials. As discussed in Section 3, the van der Waals force is the main reason that keeps carbon nanomaterials gathered. However, the functionalization treatment is an effective method to achieve homogeneous distributions [102,103] and is considered as one of the best methods to prevent the aggregation of nanofillers [33]. The functionalization treatment has two kinds: covalent functionalization and non-covalent functionalization. Both kinds are based on alteration of the bond connectivity. Covalent functionalization is to form a covalent linkage between the functional units and the skeleton of CNTs or graphene. Such linkage could achieve a high quality of functionalization, but the shortcoming with this method is that it can destroy the translational symmetry of the CNTs and graphene, which may lead to a significant change in the electrical and mechanical properties. Non-covalent functionalization is to form the bond connectivity of functional units and CNTs or graphene without destroying the π–π conjugation [104]. This could preserve the physical properties of CNTs or graphene, but the shortcoming is that functionalization is not stable [33]. As both methods of functionalization have pros and cons, the choice of them is highly case by case in specific applications. Nevertheless, there have been some experiences in choosing a particular functionalization method. In the following, a detailed discussion of covalent and non-covalent functionalization is introduced to help readers design their experiments.

**Figure 12:**
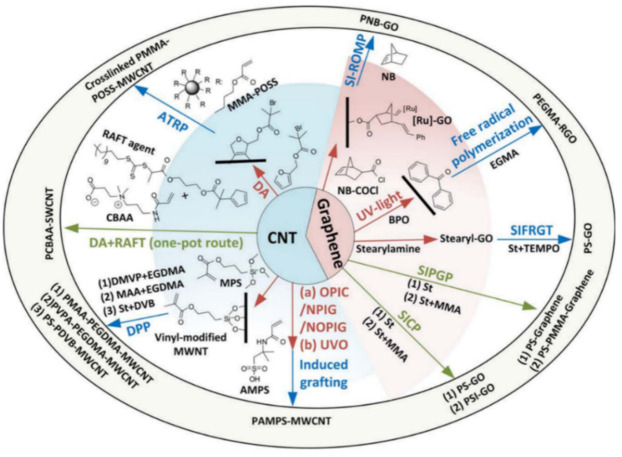
Covalent functionalization using the “grafting from” approach [105]. Reproduced with permission.

**Figure 13:**
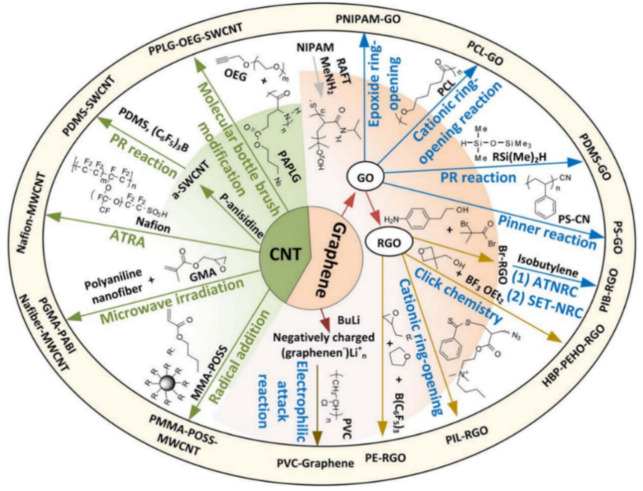
Covalent functionalization using the “grafting to” approach [105]. Reproduced with permission.

For covalent functionalization, cationic, anionic, and radical polymerizations are three different categories and the atom transfer radical polymerization is the most effective and widely used one [33]. Research has been conducted by using the atom transfer radical polymerization method to functionalize carbon nanomaterials with different polymers. Liu et al. conducted a detailed review on covalent functionalization of carbon nanomaterials with polymers. They characterized covalent functionalization into two groups based on the different approaches, “grafting from” and “grafting to”. For the “grafting from” approach, it contains three steps in the following order: introduction of the desired functional group, covalent modification, and grafting the polymer from carbon nanomaterials. For the “grafting to” approach, it firstly synthesizes polymer chains and then links the polymer chains with functional groups on the surface of carbon nanomaterials [105]. Different polymers include PEGMA [106], PS/PSI [107], PNB [108], crosslinked PMMA–POSS [109], and PCBAA [110], which are grafted from carbon nanomaterials using the “graft from” approach. Further, there has been research that used the “graft to” approach to functionalize carbon nanomaterials with polymers, including PDMS [111,112], PMMA–POSS [113], and PS [114]. Figure 12 and Figure 13 show the technical procedures of covalent functionalization of carbon nanomaterials with different polymers using the “grafting from” and “grafting to” approaches.

In the scope of tactile and thermal sensors, a good example of using covalent functionalization is Zhang et al.’s work. Tetravinyl tetramethyl cyelo tetrasiloxane-modified MWNTs were grafted with poly (vinylmethylsiloxane) (PVMS). The composite shows good piezo resistance repeatability [115].

Recently, Raimondo et al. explored new kinds of functionalization treatments that could improve the compatibility with the polymer matrix while preventing sacrificing CNTs’ extraordinary electrical and mechanical properties [116]. Covalent functionalization was performed by using 4,4′-diaminodiphenyl sulfone as the functional unit. At the same time, 4,4′-diaminodiphenyl sulfone was also employed as the matrix filler to increase the physical bonds between functionalized CNTs and the epoxy matrix. Raimondo’s method uses the same material (4,4′-diaminodiphenyl sulfone) as the functional unit and the matrix filler. It was demonstrated as an effective method to achieve better dispersion and preservation of physical properties at the same time.

For non-covalent functionalization, it preserves the extraordinary properties of carbon nanomaterials while adding new functionalities. Since π–π conjugation is maintained in the structure of carbon nanomaterials, H bonding and π–π stacking play important roles in non-covalent functionalization [117]. Non-covalent functionalization is more promising for tactile and thermal sensors considering that the function requirements for the sensor need the mechanical, electrical, and thermal properties of carbon nanomaterials. Especially for the biocompatibility property, non-covalent functionalization is preferred to establish an interaction between carbon nanomaterials and biomolecules without compromising π–π conjugation [118]. Liang et al. used non-covalent functionalization to achieve molecular-level dispersion of graphene oxide in a poly (vinyl alcohol) (PVA) matrix and the nanocomposite was found to obtain a better mechanical property. E. Choi et al. approved that reduced graphene after non-covalent functionalization could achieve stable dispersion in various organic solvents [117].

In summary, the fabrication of CNPCs is mainly determined by two essential factors: the dispersion of carbon nanomaterials in a designated solvent and the bond connectivity of carbon nanomaterials. Different fabrication methods, surfactants, and functionalization approaches can be chosen to achieve homogeneous distribution and present mechanical, electrical, and thermal properties to meet the designed function requirements.

## 7. Conclusions and the Future Direction

This paper presented a critical review of tactile and thermal sensors which are built from carbon nanomaterial-filled polymer composites (CNPC)—CNPC sensors for short. The review was assisted by a general knowledge architecture of a system, namely, FCBPSS. The review covered the principle, structure and state, behavior and performance, and fabrication of CNPC sensors. Several conclusions can be drawn from this review.

(1) The design and fabrication of a single CNPC sensor is ad hoc, i.e., far less systematic. There is no well documented knowledge available regarding the relationship of various parameters of CNPCs with respect to the performance of CNPC sensors. It is noted that the performance includes the following matrices: sensitivity, accuracy, reliability, robustness, and resilience [3,4].
(2) Networks of CNPC sensors, i.e., intelligent tactile sensing systems, are still in their infancy. Currently, there is no theory available to guide the design and fabrication of such networks and operate and manage them. It is noted that for applications such as human–robot interaction or human cooperative robotics, real-time adaption of a network of CNPC sensors is imperative because in these applications, a target system changes with respect to time and event.
(3) Both a single CNPC sensor and a network of CNPC sensors are suitable for flat surfaces only. This is an important limitation to applications such as human cooperative robotics, where a curved body surface is required.

Based on the above discussion, a few important future works are proposed with the main application in human cooperative robotics. First, for a single CNPC sensor, there is a need to develop a mathematical model for the relationship among various variables on the structure of CNPC sensors, fabrication variables, and sensor performance. The model should be applied to develop a systematical design process for CNPC sensors, which is expected to develop a CNPC for a particular requirement. It is noted that such a mathematical model could be built by combining machine learning and the knowledge about the principle along with the structure–function (sensing in this case) relation.

Second, for a network of CNPC sensors, there is a need to develop a mathematical model for the relationship of the variables, which describe the network, with respect to the performance of the network. The performance includes not only the accuracy but also the robustness and resilience of the network. It is noted that such a mathematical model may likely be built with machine learning techniques because of complexities in the network, e.g., the coupling effects between individual CNPC sensors and the network of them.

Third, it is imperative to develop a CNPC sensor that adapts physically to a curved body shape. The challenge here is about knowing changes in internal stresses and their distribution in the material system and how these changes affect the sensor performance. To probe this development, a careful mechanics analysis of a CNPC sensor when its structure changes due to its installation on a different body surface should be carried out.

Fourth, CNPCs can also be shaped as a medium between two parallel electrode plates, and the whole system can serve as a capacitive pressure sensor [119,120,121]. How the CNPC discussed in this paper, including the structure and fabrication technique, would affect the property (e.g., dialectic constant) of such a medium in a capacitive pressure sensor is worthy of study.

## Figures and Tables

**Table 1:** The properties of different carbon nanomaterials [31].

Carbon Nanomaterial	Single-Walled Carbon Nanotube	Multi-Walled Carbon Nanotube	Fullerene	Graphite	Graphene
Specific gravity (g/cm^3^)	0.8	1.8	1.7	1.9~2.3	
Electrical conductivity (S/cm)	10^2^~10^6^	10^3^~10^5^	10^−5^	4000 ^P^, 3.3 ^C^	10^3^
Thermal conductivity (W/(mK))	6000	2000	0.4	298 ^P^, 2.2 ^C^	5000
Thermal stability in air (°C)	>600	>600	~600	450~650	600

^P^: in-plane; ^C^: c-axis [31].

**Table 2:** Comparison between graphene and multi-walled carbon nanotube (MWCNT).

Filler	Purity of Fillers %		Aspect Ratio Average Length/Thickness (or ∅)
	**Carbon Purity**	**CO/Metal Oxide Groups**	
Graphene ^a^	~84	~16	~250
MWCNT ^b^	90	10	157.9

^a^: data obtained from [32]; ^b^: provided by NanocylTM company.

**Table 3:** The electrical conductivity and percolation threshold of carbon nanotube/graphene-based polymer nanocomposites.

Filler	Matrix	Filler Loading	Fabrication Method	Percolation Threshold	Highest Electrical Conductivity (S/m)	Reference
MWCNT	Polyphenylene sulfide		Melt mixing	5 wt%	-	[36]
Graphene	Polyphenylene sulfide		Melt mixing	10 wt%	-	[36]
MWCNT	Epoxy	2 wt%	Milling	0.2 wt%	1.76 × 10^−1^	[37]
Graphene	Epoxy	2 wt%	Milling	0.2 wt%	4.0 × 10^−3^	[37]
MWCNT	Polyetherimide	5 wt%	Solution mixing	-	1.43 × 10^−4^	[38]
Graphene	Polyetherimide	5 wt%	Solution mixing	0.22 wt%	5.82 × 10^−4^	[38]
Graphene/MWCNT	Polyetherimide	5 wt%	Solution mixing	-	1.28 × 10^−3^	[38]
MWCNT	Polydimethylsiloxane	4 wt%	Solution mixing	-	2.53 × 10^−5^	[39]
Graphene	Polydimethylsiloxane	4 wt%	Solution mixing	-	7.89 × 10^−5^	[39]
Graphene/MWCNT	Polydimethylsiloxane	4 wt%	Solution mixing	-	1.24 × 10^−3^	[39]
MWCNT	High density polyethylene	-	Alcohol-assisted dispersion and hot pressing	0.25 vol%	-	[40]
Graphene	High density polyethylene	-	Alcohol-assisted dispersion and hot pressing	1 vol%	-	[40]
MWCNT	Polystyrene/ poly (2,6-dimethyl-1,4-phenylene oxide)	4 wt%	Solution blending	0.2 wt%	57	[32]
Graphene	Polystyrene/ poly (2,6-dimethyl-1,4-phenylene oxide)	4 wt%	Solution blending	1 wt%	0.9	[32]
CNT	Polyaniline	69.2 wt%	In situ polymerization	-	680	[41]
Graphene	Polyaniline	69.2 wt%	In situ polymerization	-	150	[41]
CNT/Graphene	Polyaniline	69.2 wt%	In situ polymerization	-	410	[41]
MWCNT	Polystyrene	5 wt%	Melt mixing	0.05 wt%	7.98 × 10^−1^	[42]
Reduced GO	PolystyrenePS	4 wt%	Solution mixing	-	22.68	[43]
CTAB/wrapped GO	Poly (vinyl chloride)	6.47 vol%	Solution mixing	0.6 vol%	5.8	[44]
MWCNT	Poly (vinyl chloride)	20 wt%	Solution mixing	-	175	[45]
MWCNT	Liquid crystalline polymer	4 wt%	Melt mixing	-	1.3 × 310^−1^	[46]
Graphene	Liquid crystalline polymerLCP	5 wt%	Solution casting and compression molding	3 wt%	4.5 × 10^−1^	[47]
MWCNT	Poly(styrene–butadiene–styrene) SBS	5 wt%	Solution mixing	0.35 wt%		[48]

**Table 4:** Suitable application of different polymers as the sensor matrix.

Polymer Type	Suitable Application as the Sensor Matrix
Thermosets	Tactile sensor
Thermoplastics	Thermal sensor
Elastomers	Tactile sensor with high sensitivity
Fibers	Tactile and thermal sensor

**Table 5:** Electrical percolation thresholds for CNT-filled thermoplastic and thermoset polymers [35].

Polymer Type	Electrical Percolation Thresholds (CNT)
Thermosets	0.1 to 1 wt%
Thermoplastics	0.2 to 15 wt%

**Table 6:** Several tactile sensing systems.

Structure	Filler	Matrix	Filler Loading	Performance	Reference
Single sensor	Graphene	Polysilicon	6.8 vol%	Gauge factor = 535	[71]
Single sensor	Carbon black	SEBS–Block copolymer	50 wt%	Gauge factor = 20	[72]
Single sensor	MWCNT	Polysulfone	0.5 wt%	Gauge factor = 2.78	[73]
Band	Graphene	Rubber	0.2 vol%	Gauge factor = 35	[74]
Band	Reduced graphene oxide	VHB elastomer		S = $\frac{\Delta R/R}{\Delta P}$= 1.37 kPa^−1^	[75]
5 × 5 array	MWCNT	Polydimethylsiloxane	7 wt%	ΔR/R0 = 0.6	[76]
16 × 17 array	Carbon black	Polymer foam			[77]
6 × 8 array	MWCNT	Polydimethylsiloxane	6 wt%	S = $\frac{\Delta R/R}{\Delta P}$= 16.9 kPa^−1^	[78]
Serpentine structures	Carbon black	Polydimethylsiloxane	25 wt%	Gauge factor = 29.1	[79]
11 × 11 array	MWCNT	Thermoplastic Polyurethane	11.1 wt%	Gauge factor = 2800	[80]
Rosette-type	Carbon black	Polydimethylsiloxane	15 wt%		[81]
8 × 8 × 2		Conductive polymer			[82]
5 paddings	graphite	Rubber			[83]
14 lines	MWCNT	Acrylate monomer	1 wt%	-	[84]

**Table 7:** Several thermal sensing systems.

Structure	Filler	Matrix	Filler Loading	Temperature Resistance Effect	Performance	Reference
Single sensor	CNT	Polyethylene	50 wt%	NTC	Linear between 25 and 45 °C	[85]
Single sensor	SWCNT	Polymer based on hydrogen bonds	20 wt%	NTC	Linear between 0 and 40 °C	[25]
Single sensor	MWCNT	Bisphenol-F epoxy resin	3 wt%	PTC	64 Ω m K^−1^	[86]
Single sensor	MWCNT	DiGlycidil-Ether Bisphenol-A/4,4-diaminodiphenyl sulfone	1 wt%	NTC	Linear between 30 and 110 °C	[87]
Single sensor	MWCNT	high-density polyethylene	5.4 wt%	PTC		[88]
Single sensor	MWCNT	DiGlycidil-Ether Bisphenol-A/4,4-diaminodiphenyl sulfone	0.5 wt%	NTC	Temperature range between room temperature and 150 °C	[23]
Single sensor	SWCNT	Polystyrene	2 wt%	NTC	7 × 10^−7^ Ω m K^−1^	[89]

**Table 8:** Advantages, disadvantages, and notes of different fabrication methods.

Fabrication Methods	Advantages	Disadvantages	Fabrication Notes
Solution method	Help distributes fillers homogeneously	Complicated procedures	Need appropriate solvent
Melt mixing method	Easy procedures	Not easy to distribute fillers homogeneously	Not appliable to thermosets
In situ polymerization method	Easy procedures	Only applies to certain polymers	Better with functionalization of carbon nanomaterials and surfactants

## References

[1] Modi S., Lin Y., Cheng L., Yang G., Liu L., Zhang W. (2011). A socially inspired framework for human state inference using expert opinion integration. IEEE/ASME Trans. Mechatron..

[2] Lin Y. (2017). Toward Intelligent Human Machine Interactions. Mech. Eng..

[3] Zhang W., Lin Y. (2010). On the principle of design of resilient systems—Application to enterprise information systems. Enterp. Inf. Syst..

[4] Zhang W., Van Luttervelt C. (2011). Toward a resilient manufacturing system. CIRP Ann. Manuf. Technol..

[5] Miao Y., Yang Q.Q., Sammynaiken R., Zhang W.J., Maley J., Schatte G. (2013). Influence of aligned carbon nanotube networks on piezoresistive response in carbon nanotube films under in-plane straining. Appl. Phys. Lett..

[6] Miao Y., Yang Q., Chen L., Sammynaiken R., Zhang W.J. (2012). Modelling of piezoresistive response of carbon nanotube network-based films under in-plane straining by percolation theory. Appl. Phys. Lett..

[7] Choudhary N., Hwang S., Choi W. (2014). Carbon Nanomaterials: A Review. Handbook of Nanomaterials Properties.

[8] Briscoe B.J., Sinha S.K., Swallowe G.M. (1999). Hardness and Normal Indentation of Polymers. Mechanical Properties and Testing of Polymers.

[9] Miao Y., Chen L., Sammynaiken R., Lin Y., Zhang W.J. (2011). Note: Optimization of piezoresistive response of pure carbon nanotubes networks as in-plane strain sensors. Rev. Sci. Instrum..

[10] Lin Y., Zhang W. (2005). A function-behavior-state approach to designing human-machine interface for nuclear power plant operators. IEEE Trans. Nucl. Sci..

[11] Zhang W., Wang J.W., Zhang W., Wang J.W. (2016). Design theory and methodology for enterprise systems. Enterp. Inf. Syst..

[12] Lin Y., Zhang W. (2004). Towards a novel interface design framework: Function-behavior-state paradigm. Int. J. Hum. Comput. Stud..

[13] Zhang W., Lin Y., Sinha N. On the function-behavior-structure model for design. Proceedings of the Canadian Design Engineering Network (CDEN) Conference.

[14] Miao Y. (2013). On Understanding of Piezoresistive Response in Carbon Nanotube Networks under In-Plane Straining. Ph.D. Thesis.

[15] Weng W., Chen G.-H., Wu D.-J., Yan W.-L. (2004). HDPE/expanded graphite electrically conducting composite. Compos. Interfaces.

[16] Zheng W., Wong S.-C. (2003). Electrical conductivity and dielectric properties of PMMA/expanded graphite composites *Compos*. Sci. Technol..

[17] Chen X.-M., Shen J.-W., Huang W.-Y. (2002). Novel electrically conductive polypropylene/graphite nanocomposites. J. Mater. Sci. Lett..

[18] Yue L., Pircheraghi G., Monemian S.A., Manas-Zloczower I. (2014). Epoxy composites with carbon nanotubes and graphene nanoplatelets—Dispersion and synergy effects. Carbon..

[19] Feng X., Liao G., He W., Sun Q., Jian X., Du J. (2009). Preparation and characterization of functionalized carbon nanotubes/poly(phthalazinone ether sulfone ketone)s composites. Polym. Compos..

[20] Xie H., Sheng P. (2009). Fluctuation-induced tunneling conduction through nanoconstrictions. Phys. Rev. B Condens. Matter Mater. Phys..

[21] Xi Y., Ishikawa H., Bin Y., Matsuo M. (2004). Positive temperature coefficient effect of LMWPE-UHMWPE blends filled with short carbon fibers. Carbon..

[22] Ferrara M., Neitzert H., Sarno M., Gorrasi G., Sannino D., Vittoria V., Ciambelli P. (2007). Influence of the electrical field applied during thermal cycling on the conductivity of LLDPE/CNT composites. Phys. E Low Dimens. Syst. Nanostruct..

[23] Neitzert H.C., Vertuccio L., Sorrentino A. (2011). Epoxy/MWCNT composite as temperature sensor and Electrical heating element. IEEE Trans. Nanotechnol..

[24] Bao S.P., Liang G.D., Tjong S.C. (2009). Positive temperature coefficient effect of polypropylene/carbon nanotube/montmorillonite hybrid nanocomposites. IEEE Trans. Nanotechnol..

[25] Yang H., Qi D., Liu Z., Chandran B.K., Wang T., Yu J., Chen X. (2016). Soft Thermal Sensor with Mechanical Adaptability. Adv. Mater..

[26] Yang S.Y., Lin W.N., Huang Y.L., Tien H.W., Wang J.Y., Ma C.C.M., Li S.M., Wang Y.S. (2011). Synergetic effects of graphene platelets and carbon nanotubes on the mechanical and thermal properties of epoxy composites. Carbon..

[27] Grossiord N., Loos J., Regev A.O., Koning C.E. (2006). Toolbox for dispersing carbon nanotubes into polymers to get conductive nanocomposites. Chem. Mater..

[28] Roy S., Petrova R.S., Mitra S. (2018). Effect of carbon nanotube (CNT) functionalization in epoxy-CNT composites. Nanotechnol. Rev..

[29] Pötschke P., Dudkin S.M., Alig I. (2003). Dielectric spectroscopy on melt processed polycarbonate—Multiwalled carbon nanotube composites. Polymer.

[30] Wang X., Tang F., Cao Q., Qi X., Pearson M., Li M., Pan H., Zhang Z., Lin Z. (2020). Comparative study of three carbon additives: Carbon nanotubes, graphene, and fullerene-c60, for synthesizing enhanced polymer nanocomposites. Nanomaterials.

[31] Ma P.-C., Siddiqui N.A., Marom G., Kim J.-K. (2010). Dispersion and functionalization of carbon nanotubes for polymer-based nanocomposites: A review. Compos. Part A Appl. Sci. Manuf..

[32] Ghislandi M.M., Tkalya E.E., Schillinger S., Koning C.E., De With G. (2013). High performance graphene- and MWCNTs-based PS/PPO composites obtained via organic solvent dispersion. Compos. Sci. Technol..

[33] Punetha V.D., Rana S., Yoo H.J., Chaurasia A., Mcleskey J.T., Ramasamy M.S., Sahoo N.G., Cho J.W. (2017). Functionalization of carbon nanomaterials for advanced polymer nanocomposites: A comparison study between CNT and graphene. Prog. Polym. Sci..

[34] Zhang W., Ouyang P., Sun Z. A Novel Hybridization Design Principle for Intelligent Mechatronics Systems. https://www.jstage.jst.go.jp/article/jsmeicam/2010.5/0/2010.5_67/_article#citedby-wrap.

[35] Min C., Shen X., Shi Z., Chen L., Xu Z. (2010). The electrical properties and conducting mechanisms of carbon nanotube/polymer nanocomposites: A review. Polym. Plast. Technol. Eng..

[36] Khan M.O., Leung S.N., Chan E., Naguib H., Dawson F., Adinkrah V. (2013). Effects of microsized and nanosized carbon fillers on the thermal and electrical properties of polyphenylene sulfide-based composites. Polym. Eng. Sci..

[37] He Z., Zhang X., Chen M., Li M., Gu Y., Zhang Z., Li Q. (2013). Effect of the filler structure of carbon nanomaterials on the electrical, thermal, and rheological properties of epoxy composites. J. Appl. Polym. Sci..

[38] Kong K., Mariatti M., Rashid A., Busfield J. (2014). Enhanced conductivity behavior of polydimethylsiloxane (PDMS) hybrid composites containing exfoliated graphite nanoplatelets and carbon nanotubes. Compos. Part B Eng..

[39] Kumar S., Sun L.L., Caceres S., Li B., Wood W., Perugini A., Maguire R.G., Zhong W.H. (2010). Dynamic synergy of graphitic nanoplatelets and multi-walled carbon nanotubes in polyetherimide nanocomposites. Nanotechnology.

[40] Du J., Zhao L., Zeng Y., Zhang L., Li F., Liu P., Liu C. (2011). Comparison of electrical properties between multi-walled carbon nanotube and graphene nanosheet/high density polyethylene composites with a segregated network structure. Carbon.

[41] Lu X., Dou H., Yang S., Hao L., Zhang L., Shen L., Zhang F., Zhang X. (2011). Fabrication and electrochemical capacitance of hierarchical graphene/polyaniline/carbon nanotube ternary composite film. Electrochim. Acta.

[42] Knapp B., Kohl P.A. (2014). Polymers for microelectronics. J. Appl. Polym. Sci..

[43] Wu N., She X., Yang D., Wu X., Su F., Chen Y. (2012). Synthesis of network reduced graphene oxide in polystyrene matrix by a two-step reduction method for superior conductivity of the composite. J. Mater. Chem..

[44] Vadukumpully S., Paul J., Mahanta N., Valiyaveettil S. (2011). Flexible conductive graphene/poly (vinyl chloride) composite thin films with high mechanical strength and thermal stability. Carbon.

[45] Broza G., Piszczek K., Schulte K., Sterzyński T. (2007). Nanocomposites of poly (vinyl chloride) with carbon nanotubes (CNT). Compos. Sci. Technol..

[46] Sahoo N.G., Cheng H.K.F., Bao H., Li L., Chan S.H., Zhao J. (2011). Nitrophenyl functionalization of carbon nanotubes and its effect on properties of MWCNT/LCP composites. Macromol. Res..

[47] Biswas S., Fukushima H., Drzal L.T. (2011). Mechanical and electrical property enhancement in exfoliated graphene nanoplatelet/liquid crystalline polymer nanocomposites. Compos. Part A Appl. Sci. Manuf..

[48] Qin B., Li B., Zhang J., Xie X., Li W. (2020). Highly sensitive strain sensor based on stretchable sandwich-type composite of carbon nanotube and poly(styrene–butadiene–styrene). Sens. Actuators A Phys..

[49] Pradhan B., Srivastava S.K. (2014). Synergistic effect of three-dimensional multi-walled carbon nanotube-graphene nanofiller in enhancing the mechanical and thermal properties of high-performance silicone rubber. Polym. Int..

[50] Zhu J., Peng H., Rodriguez-Macias F., Margrave J.L., Khabashesku V.N., Imam A.M., Lozano K., Barrera E.V. (2004). Reinforcing epoxy polymer composites through covalent integration of functionalized nanotubes. Adv. Funct. Mater..

[51] DeLozier D.M., Watson K.A., Smith J.G., Clancy T.C., Connell J.W. (2006). Investigation of aromatic/aliphatic polyimides as dispersants for single wall carbon nanotubes. Macromolecules.

[52] Du F., Guthy C., Kashiwagi T., Fischer J.E., Winey K.I. (2006). An infiltration method for preparing single-wall nanotube/ epoxy composites with improved thermal conductivity. J. Polym. Sci. Part B Polym. Phys..

[53] Li J., Ma P.C., Chow W.S., To C.K., Tang B.Z., Kim J.-K. (2007). Correlations between percolation threshold, dispersion state, and aspect ratio of carbon nanotubes. Adv. Funct. Mater..

[54] Stauffer D., Bunde A. (1993). Introduction to Percolation Theory. Phys. Today.

[55] Regev O., ElKati P.N.B., Loos J.J., Koning C.E. (2004). Preparation of conductive nanotube-polymer composites using latex technology. Adv. Mater..

[56] Martin C.A., Sandler J.K.W., Shaffer M.S.P., Schwarz M.K., Bauhofer W., Schulte K., Windle A.H. (2004). Formation of percolating networks in multi-wall carbon-nanotube-epoxy composites. Compos. Sci. Technol..

[57] Sandler J., Kirk J.E., Kinloch I.A., Shaffer M., Windle A.H. (2003). Ultra-low electrical percolation threshold in carbon-nanotube-epoxy composites. Polymer.

[58] Hu G., Zhao C., Zhang S., Yang M., Wang Z. (2006). Low percolation thresholds of electrical conductivity and rheology in poly(ethylene terephthalate) through the networks of multi-walled carbon nanotubes. Polymer.

[59] Liao S.H., Yen C.Y., Weng C.C., Lin Y.F., Ma C.C.M., Yang C.H., Tsai M.C., Yen M.Y., Hsiao M.C., Lee S.J. (2008). Preparation and properties of carbon nanotube/polypropylene nanocomposite bipolar plates for polymer electrolyte membrane fuel cells. J. Power Sources.

[60] JEngel J., Chen J., Chen N., Pandya S., Liu C. Multi-Walled Carbon Nanotube Filled Conductive Elastomers: Materials and Application to Micro Transducers. Proceedings of the 19th IEEE International Conference on Micro Electro Mechanical Systems.

[61] Najer A., Wu D., Vasquez D., Palivan C.G., Meier W. (2013). Polymer nanocompartments in broad-spectrum medical applications. Nanomedicine.

[62] Zhang H., Patel P.R., Xie Z., Swanson S.D., Wang X., Kotov N.A. (2013). Tissue-compliant neural implants from microfabricated carbon nanotube multilayer composite. ACS Nano.

[63] Tsang W., Stone A., Aldworth Z., Otten D., Akinwande A., Daniel T. Remote Control of a Cyborg Moth Using Carbon Nanotube-enhanced Flexible Neuroprosthetic Probe. Proceedings of the 2010 IEEE 23rd International Conference on Micro Electro Mechanical Systems (MEMS).

[64] Kim D.-H., Ghaffari R., Lu N., Rogers J.A. (2012). Flexible and Stretchable Electronics for Biointegrated Devices. Annu. Rev. Biomed. Eng..

[65] David-Pur M., Bareket-Keren L., Beit-Yaakov G., Raz-Prag D., Hanein Y. (2014). All-carbon-nanotube flexible multi-electrode array for neuronal recording and stimulation. Biomed. Microdevices.

[66] David-Pur M., Bareket-Keren L., Beit-Yaakov G., Raz-Prag D., Rand D., Hanein Y., Rand D. Carbon-nanotube based flexible electrodes for retinal recording and stimulation. Proceedings of the IEEE Sensors.

[67] Zhang H., Zhou L., Zhang W. (2014). Control of scaffold degradation in tissue engineering: A review. Tissue Eng. Part B Rev..

[68] Yin R., Zhang N., Wang K., Long H., Xing T., Nie J., Zhang H., Zhang W. (2017). Material design and photo-regulated hydrolytic degradation behavior of tissue engineering scaffolds fabricated via 3D fiber deposition. J. Mater. Chem. B.

[69] Armentano I., Dottori M., Fortunati E., Mattioli S., Kenny J. (2010). Biodegradable polymer matrix nanocomposites for tissue engineering: A review. Polym. Degrad. Stab..

[70] Guo Y., Chang C.C., Halada G., Cuiffo M.A., Xue Y., Zuo X., Pack S., Zhang L., He S., Weil E. (2017). Engineering flame retardant biodegradable polymer nanocomposites and their application in 3D printing. Polym. Degrad. Stab..

[71] Boland C.S., Khan U., Ryan G., Barwich S., Charifou R., Harvey A., Backes C., Li Z., Ferreira M.S., Möbius M.E. (2016). Sensitive electromechanical sensors using viscoelastic graphene-polymer nanocomposites. Science.

[72] Mattmann C., Clemens F., Tröster G. (2008). Sensor for measuring strain in textile. Sensors.

[73] Oliva-Avilés A.I., Avilés F., Sosa V. (2011). Electrical and piezoresistive properties of multi-walled carbon nanotube/polymer composite films aligned by an electric field. Carbon.

[74] Boland C.S., Khan U., Backes C., O'Neill A., Mccauley J., Duane S., Shanker R., Liu Y., Jurewicz I., Dalton A.B. (2014). Sensitive, high-strain, high-rate bodily motion sensors based on graphene-rubber composites. ACS Nano.

[75] Chang T.H., Tian Y., Li C., Gu X., Li K., Yang H., Sanghani P., Lim C.M., Ren H., Chen P.Y. (2019). Stretchable Graphene Pressure Sensors with Shar-Pei-like Hierarchical Wrinkles for Collision-Aware Surgical Robotics. ACS Appl. Mater. Interfaces.

[76] Kong J.-H., Jang N.-S., Huh J.-Y., Kim S.-H., Kim J.-M. (2015). Piezoresistive polymer diaphragm sensor array using conductive elastomeric nanocomposite films for skin-mountable keypad applications. J. Microelectromech. Syst..

[77] Goger D., Worn H. (2007). A highly versatile and robust tactile sensing system. Proc. IEEE Sens..

[78] Sun X., Wang C., Chi C., Xue N., Liu C. (2018). A highly-sensitive flexible tactile sensor array utilizing piezoresistive carbon nanotube–polydimethylsiloxane composite. J. Micromech. Microeng..

[79] Lu N., Lu C., Yang S., A Rogers J. (2012). Highly sensitive skin-mountable strain gauges based entirely on elastomers. Adv. Funct. Mater..

[80] He Z., Zhou G., Byun J.H., Lee S.K., Um M.K., Park B., Kim T., Lee S.B., Chou T.W. (2019). Highly stretchable multi-walled carbon nanotube/thermoplastic polyurethane composite fibers for ultrasensitive, wearable strain sensors. Nanoscale.

[81] Kong J.-H., Jang N.-S., Kim S.-H., Kim J.-M. (2014). Simple and rapid micropatterning of conductive carbon composites and its application to elastic strain sensors. Carbon.

[82] Cheng M.-Y., Tsao C.-M., Lai Y.-Z., Yang Y.-J. (2011). The development of a highly twistable tactile sensing array with stretchable helical electrodes. Sens. Actuators A Phys..

[83] Chen Y., Yu M., Bruck H.A., Smela E. (2016). Stretchable touch-sensing skin over padding for co-robots. Smart Mater. Struct..

[84] Vatani M., Engeberg E.D., Choi J.W. (2013). Force and slip detection with direct-write compliant tactile sensors using multi-walled carbon nanotube/polymer composites. Sens. Actuators A Phys..

[85] Karimov K.S., Abid М., Saleem M., Akhmedov K.M., Bashir M.M., Shafique U., Ali M.M. (2014). Temperature gradient sensor based on CNT composite. Phys. B Condens. Matter..

[86] Li Y., Hu N., Wu L., Yuan W., Peng X., Gu B., Chang C., Liu Y., Ning H., Li J. (2013). Temperature-dependent piezoresistivity in an MWCNT/epoxy nanocomposite temperature sensor with ultrahigh performance. Nanotechnology.

[87] Lamberti P., Vivo B., Spinelli G., Tucci V., Guadagno L., Raimondo M., Vertuccio L. (2016). Analysis of the Effects of Hydrotalcite Inclusion on the Temperature-Sensing Properties of CNT-Epoxy Nanocomposites. IEEE Sens. J..

[88] He X.J., Du J.H., Ying Z., Cheng H.M., He X.J. (2005). Positive temperature coefficient effect in multiwalled carbon nanotube/high-density polyethylene composites. Appl. Phys. Lett..

[89] Aliev A.E. (2008). Bolometric detector on the basis of single-wall carbon nanotube/polymer composite. Infrared Phys. Technol..

[90] Alamusi N., Hu J., Qiu Y., Li C., Chang S., Atobe H., Fukunaga Y., Liu H., Ning L., Wu J. (2013). Multi-scale numerical simulations of thermal expansion properties of CNT-reinforced nanocomposites. Nanoscale Res. Lett..

[91] Amjadi M., Kyung K.-U., Park I., Sitti M. (2016). Stretchable, Skin-Mountable, and Wearable Strain Sensors and Their Potential Applications: A Review. Adv. Funct. Mater..

[92] Yan C., Wang J., Kang W., Cui M., Wang X., Foo C.Y., Chee K.J., Lee P.S. (2014). Graphene: Highly Stretchable Piezoresistive Graphene-Nanocellulose Nanopaper for Strain Sensors. Adv. Mater..

[93] Patel S., Park H., Bonato P., Chan L., Rodgers M. (2012). A Review of Wearable Sensors and Systems with Application in Rehabilitation. J. Neuro Eng. Rehabil..

[94] Muth J.T., Vogt D.M., Truby R.L., Mengüç Y., Kolesky D.B., Wood R.J., Lewis J.A. (2014). Embedded 3D printing of strain sensors within highly stretchable elastomers. Adv. Mater..

[95] Hu N., Masuda Z., Yamamoto G., Fukunaga H., Hashida T., Qiu J. (2008). Effect of fabrication process on electrical properties of polymer/multi-wall carbon nanotube nanocomposites. Compos. Part A Appl. Sci. Manuf..

[96] Chi C., Sun X., Xue N., Li T., Liu C. (2018). Recent progress in technologies for tactile sensors. Sensors.

[97] Liu C.-X., Choi J.-W. (2012). Improved Dispersion of Carbon Nanotubes in Polymers at High Concentrations. Nanomaterials.

[98] Subramanyam U., Kennedy J.P. (2009). PVA networks grafted with PDMS branches. J. Polym. Sci. Part A Polym. Chem..

[99] Hwang J., Jang J., Hong K., Kim K.N., Han J.H., Shin K., Park C.E. (2011). Poly(3-hexylthiophene) wrapped carbon nanotube/poly(dimethylsiloxane) composites for use in finger-sensing piezoresistive pressure sensors. Carbon.

[100] Hong J., Lee J., Hong C.K., Shim S.E. (2010). Effect of dispersion state of carbon nanotube on the thermal conductivity of poly(dimethyl siloxane) composites. Curr. Appl. Phys..

[101] Chua T., Mariatti M., Aziz A., Rashid A. (2010). Effects of surface-functionalized multi-walled carbon nanotubes on the properties of poly(dimethyl siloxane) nanocomposites. Compos. Sci. Technol..

[102] Wang S. (2009). Optimum degree of functionalization for carbon nanotubes. Curr. Appl. Phys..

[103] Xu Y., Liu Z., Zhang X., Wang Y., Tian J., Huang Y., Ma Y., Zhang X., Chen Y. (2009). A graphene hybrid material covalently functionalized with porphyrin: Synthesis and optical limiting property. Adv. Mater..

[104] Das M., Ghosh S., Roy S. (2018). Non-covalent functionalization of CNTs with polycarbazole: A chemiresistive humidity sensor with tunable chemo-electric attributes at room temperature. New J. Chem..

[105] Liu J., Ye Y., Xue Y., Xie X., Mai Y.-W. (2017). Recent advances in covalent functionalization of carbon nanomaterials with polymers: Strategies and perspectives. J. Polym. Sci. Part A Polym. Chem..

[106] Roppolo I., Chiappone A., Bejtka K., Celasco E., Chiodoni A., Giorgis F., Sangermano M., Porro S. (2014). A powerful tool for graphene functionalization: Benzophenone mediated UV-grafting. Carbon.

[107] Li B., Hou W., Sun J., Jiang S., Xu L., Li G., Memon M.A., Cao J., Huang Y., Bielawski C.W. (2015). Tunable functionalization of graphene oxide sheets through surface-initiated cationic polymerization. Macromolecules.

[108] Zhang Q., Li Q., Xiang S., Wang Y., Wang C., Jiang W., Zhou H., Yang Y., Tang J. (2014). Covalent modification of graphene oxide with polynorbornene by surface-initiated ring-opening metathesis polymerization. Polymer.

[109] Zhang W., Zhou Z., Li Q., Chen G.X. (2014). Controlled dielectric properties of polymer composites from coating multiwalled carbon nanotubes with octa-acrylate silsesquioxane through Diels-Alder cycloaddition and atom transfer radical polymerization. Ind. Eng. Chem. Res..

[110] Yameen B., Rodriguez-emmenegger C., Ahmed I., Preuss C.M., Drr C.J., Zydziak N., Trouillet V., Fruk L., Barner-kowollik C. (2013). A facile one-pot route to poly(carboxybetaine acrylamide) functionalized SWCNTs. Chem. Commun..

[111] Chadwick R.C., Grande J.B., Brook M.A., Adronov A. (2014). Functionalization of single-walled carbon nanotubes via the Piers-Rubinsztajn reaction. Macromolecules.

[112] Zhang J., Liang S., Yu L., Ladegaard Skov A., Etmimi H.M., Mallon P.E., Adronov A., Brook M.A. (2016). Silicone-modified graphene oxide fillers via the Piers-Rubinsztajn reaction. J. Polym. Sci. Part A Polym. Chem..

[113] Sun D., Zhou Z., Chen G.-X., Li Q. (2014). Regulated dielectric loss of polymer composites from coating carbon nanotubes with a cross-linked silsesquioxane shell through free-radical polymerization. ACS Appl. Mater. Interfaces.

[114] McGrail B.T., Rodier B.J., Pentzer E.B. (2014). Rapid functionalization of graphene oxide in water. Chem. Mater..

[115] Zhang X., Qasim K., Hu S. (2014). Enhanced piezoresistance repeatability of carbon nanotube/silicane composites achieved using radiation-induced graft polymerization. J. Polym. Res..

[116] Raimondo M., Naddeo C., Vertuccio L., Bonnaud L., Dubois P., Binder W.H., Sorrentino A., Guadagno L. (2020). Multifunctionality of structural nanohybrids: The crucial role of carbon nanotube covalent and non-covalent functionalization in enabling high thermal, mechanical and self-healing performance. Nanotechnology.

[117] Choi E.Y., Han T.H., Hong J., Kim J.E., Lee S.H., Kim H.W., Kim S.O. (2010). Noncovalent functionalization of graphene with end-functional polymers. J. Mater. Chem..

[118] Zhou Y., Fang Y., Ramasamy R.P. (2019). Non-covalent functionalization of carbon nanotubes for electrochemical biosensor development. Sensors.

[119] Guo X., Huang Y., Cai X., Liu C., Liu P. (2017). Capacitive wearable tactile sensor based on smart textile substrate with carbon black/silicone rubber composite dielectric. Meas. Sci. Technol..

[120] Hu C.F., Wang J.Y., Liu Y.C., Tsai M.H., Fang W. (2013). Development of 3D Carbon Nanotube Interdigitated Finger Electrodes on Polymer Substrate for Flexible Capacitive Sensor Application. Nanotechnology.

[121] Kanoun O., Bouhamed A., Ramalingame R., Bautista-Quijano J.R., Rajendran D., Al-Hamry A. (2021). Review on Conductive Polymer/CNTs Nanocomposites Based Flexible and Stretchable Strain and Pressure Sensors. Sensors.

